# Supplementary material for: Persistence of Metarhizium brunneum (Ascomycota: Hypocreales) in the Soil Is Affected by Formulation Type as Shown by Strain-Specific DNA Markers
Source: J Fungi (Basel). 2023 Feb 9;9(2):229. doi: 10.3390/jof9020229 (PMC9966207; doi:10.3390/jof9020229)
Supplement: Supplementary file 1 [file jof-09-00229-s001.zip › jof-2119659-supplementary.pdf]

Persistence of *Metarhizium brunneum* (Ascomycota: Hypocreales) in the soil is affected by formulation type as shown by strain-specific DNA markers.

Iker Hernández[1*], Clara Sant[1], Raquel Martínez[1], Marta Almazán[1], Marta Caminal[1], Víctor Quero[1], Mohammed El-Adak[1], Albert Casanova[1], Inmaculada Garrido-Jurado[2], Meelad Yousef-Yousef[2], Enrique Quesada-Moraga[2], José Manuel Lara[1], Carolina Fernández[1]8

[1]Futureco Bioscience. Avda. Del Cadí 19-23; 08799 Olèrdola (Barcelona), Spain.

[2]Department of Agronomy, ETSIAM, University of Córdoba, Córdoba, Spain.

*Corresponding author: iker@futurecobioscience.com12

Supplemental Material.

Supplemental Figure 1. Images of the machinery used in the different trials.

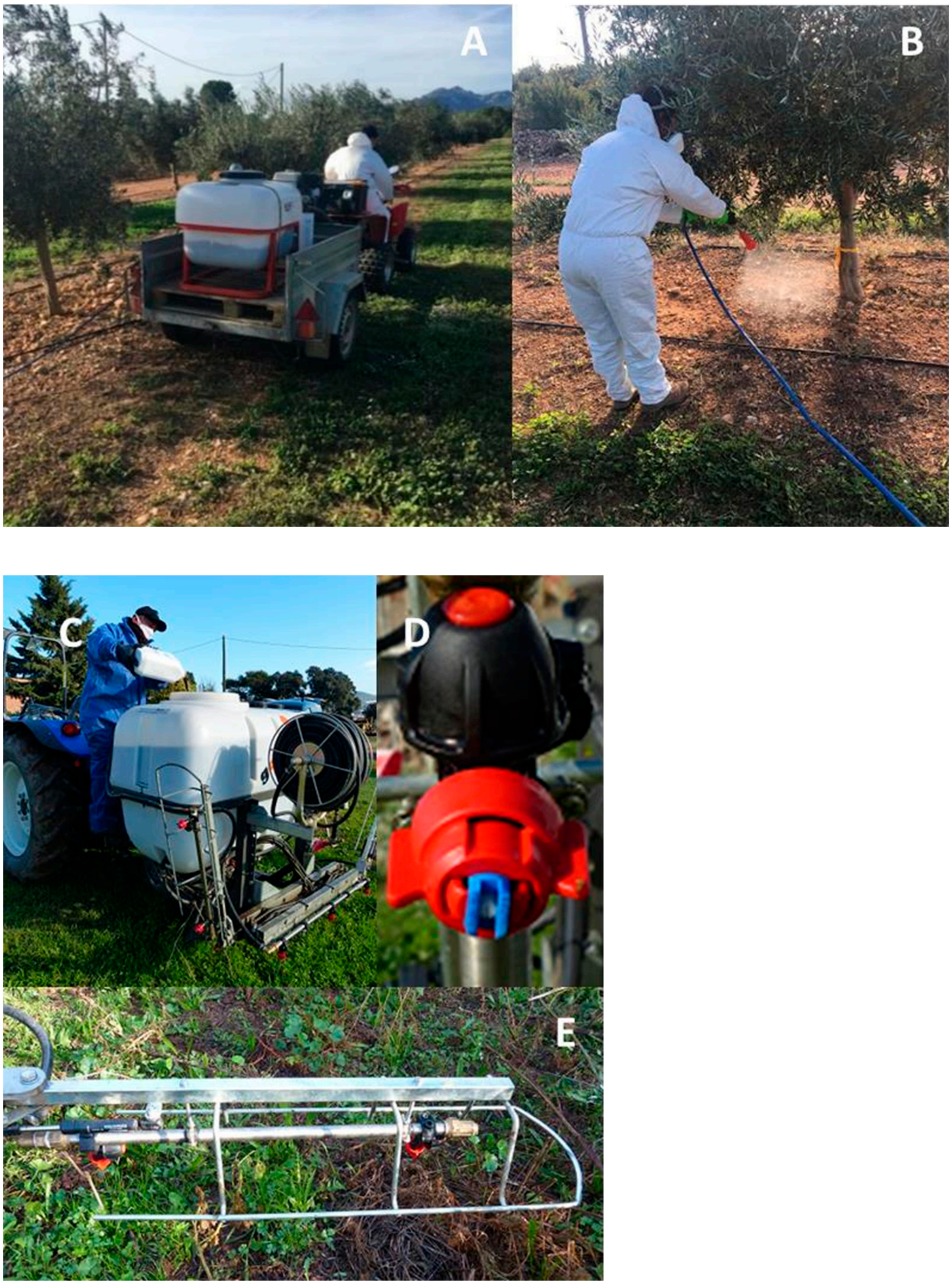

A, equipment used at Mont-roig del Camp and Avinyó Nou; B, detail of the hoss and nozzle used at Mont-roig del Camp and Avinyó Nou. C, equipment used at Caldes de

Montbui; D, detail of the nozzles used at Caldes de Montbui; E, detail of the application bar used at Caldes de Montbui under working conditions.

Supplemental Figure 2. Principal component analysis of the concentration of *Metarhizium brunneum* EAMb 09/01-Su and environmental parameters. Only the analysis for the FAM-labelled marker is shown. Data from the 2 trials at Caldes de Montbui were merged for the analysis.

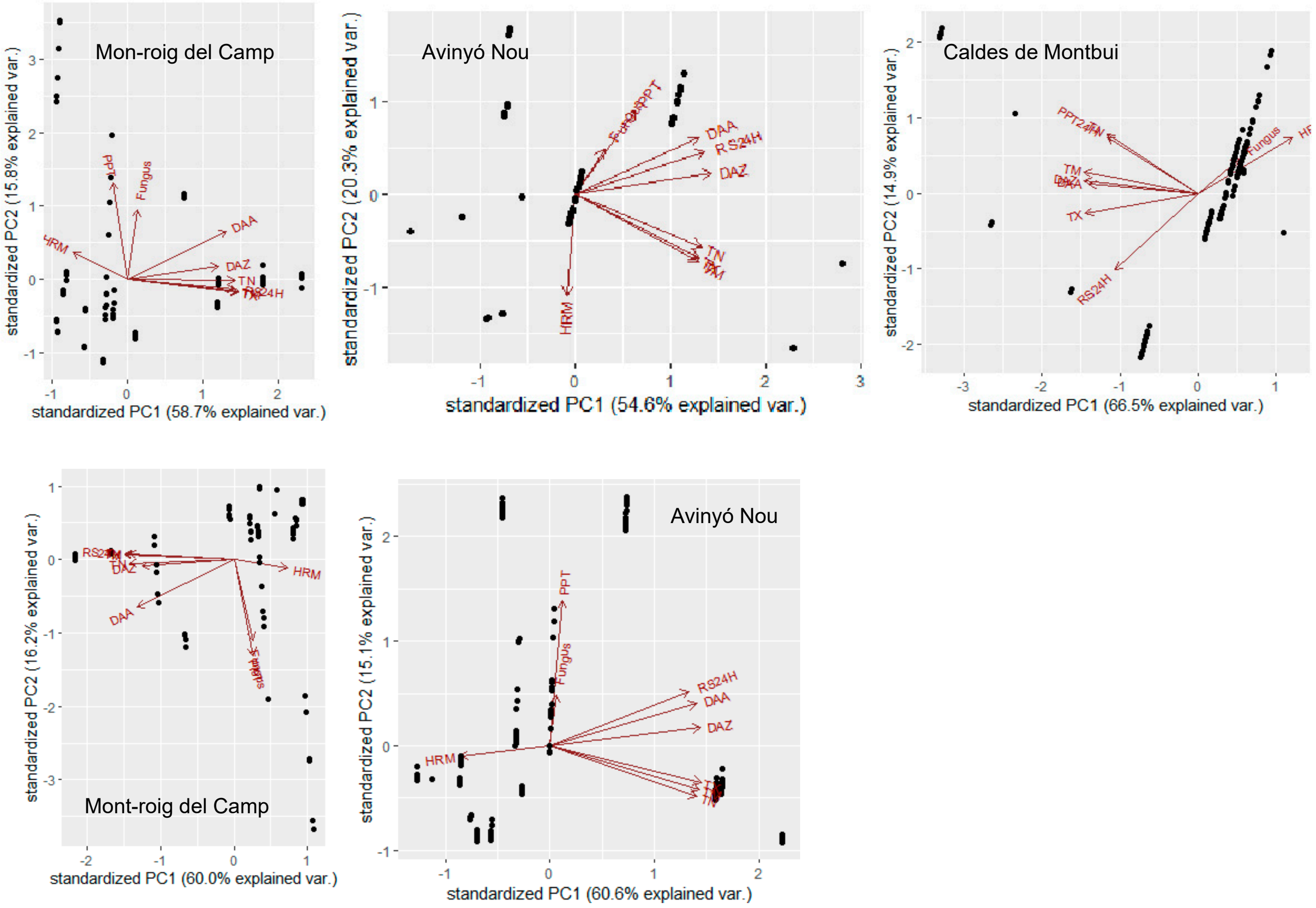

HRM, average relative humidity; TX, TN and TM, maximum, minimum and average daily temperatures, respectively; PPT24H, rainfall in the preceding 24 h; DAA and DAZ, days after the first or the last application, respectively.

3

Supplemental Table 1. *Metarhizium spp*. strains used to test primer specificity towards *Metarhizium brunneum* EAMb 09/01-Su.

Strain code	Scientific name
EAMb 09/01-	*M. brunneum*
H4	*Metarhizium anisopliae*
H5	*M. anisopliae*
H6	*M. anisopliae*
H8	*M. anisopliae*
H14	*M. anisopliae*
H221	*Metarhizium robertsii / M. anisopliae*
H339	*Metarhizium guizhouense*
H341	*Metarhizium spp.*
H399	*M. anisopliae*
H435	*M. anisopliae / M. robertsii*
H562	*M. anisopliae / M. brunneum*
H566	*M. anisopliae / M. robertsii / M. majus*
H619	*M. anisopliae / M. brunneum*
H631	*M. anisopliae / M. brunneum*
H655	*M. anisopliae / M. brunneum*
H660	*M. anisopliae / M. brunneum*
H673	*M. anisopliae / M. brunneum*
H674	*Metarhizium anisopliae*
H687	*M. anisopliae / M. brunneum*
H691	*M. anisopliae*
H701	*M. anisopliae*
H748	*M. anisopliae /M. brunneum*
H760	*M. anisopliae / M. brunneum*
H767	*M. anisopliae*
H770	*M. anisopliae / M. brunneum*

H891	*M. anisopliae*

Supplemental Table 2: Threshold cycle (C_T) values of each primer pair with the *Metarhizium brunneum* EMAb 09/01-Su strain and the other 26 *Metarhizium spp*. isolates tested.

Primer pair / Isolate	1	2	3	4	5	6	7	9	11	12	13	14	15	16	17	18	19	20	21	22
EAMb 09/01-Su	30,0	31,5	31,4	32,7	33,9	31,2	33,6	33,7	25,6	24,1	28,6	29,2	31,9	32,3	33,8	34,2	29,9	32,0	29,8	30,7
H4									34,4			31,3	32,7	34,9	33,7	35,9	30,1	32,7	29,7	31,1
H5									33,2		31,6	32,1	31,8	33,6	33,4	41,2	29,4	32,2	29,0	30,1
H6					38,0				36,0			31,8	31,7	32,7	34,7	37,5	29,1	33,1	29,5	30,6
H8		35,4							34,9		39,6	31,8	31,2	32,8	32,2	35,9	28,0	29,5	29,4	29,5
H14									38,3		40,9	30,7	31,9	33,0	34,0	36,0	29,5	32,7	29,6	30,7
H221									36,6		38,5	28,8	31,1	33,3	28,6	35,6	28,6	32,0	29,5	30,0
H339									33,2			29,5	31,1	32,2	28,1	37,5	29,2	31,9	28,9	30,1
H341		42,6	40,6						33,6		38,0	29,1	31,5	33,3	33,9	36,8	28,9	32,5	29,7	30,4
H399			39,7		41,5				35,5		37,7	32,2	31,7	32,5	34,1	33,6	27,9	31,1	29,0	29,8
H435		34,3	37,5	34,0							41,6	30,2	31,7	36,6	28,8	35,6	29,0	33,0	30,0	30,7
H562		26,3	26,9	27,7	28,5	29,5	39,2		29,1			27,6	31,0	33,4	33,5	36,7	28,7	32,2	29,4	29,9
H566		38,8	34,3			40,6					36,9	30,1	32,3	35,5	33,4		28,8	32,1	29,1	30,0
H619		38,8									39,0	34,6	31,8	34,4	33,7	35,3	28,8	32,3	29,3	30,4
H631		39,6	38,1						33,0		38,8	31,7	31,4	37,3	34,4	38,3	28,7	32,0	29,8	29,9

H655		34,7	42,5						34,1		38,6	29,6	31,9	33,0	34,3	29,2	28,7	31,7	29,6	29,6
H660			34,3			34,7			33,9			31,8	31,3	34,3	33,9	35,0	28,8	32,9	29,7	29,9
H673			42,0			35,3			34,0		37,8	31,1	30,5	32,9	33,3	36,1	28,6	32,0	28,1	29,6
H674			41,7			34,2			36,2		38,1	31,3	32,1	33,3	33,6	35,4	28,6	32,1	29,9	30,2
H687			40,8						34,6		38,6	31,6	31,7	34,1	33,5	37,0	29,5	32,5	29,8	29,5
H691		35,3	38,3			35,4			33,3		39,3	32,1	31,0	34,0	34,0	34,6	28,4	32,6	28,7	29,8
H701			40,9	34,6		35,6			37,7		39,1	32,2	31,5	33,9	33,3	35,7	29,2	32,1	30,0	29,6
H748						37,4	42,8				36,6	31,2	32,3	34,9	34,8	34,4	29,3	32,4	30,3	30,3
H760			37,9	35,5							40,7	32,7	38,0	40,5			33,5		33,5	29,7
H767			37,8			40,9							30,9	32,2	33,1	34,6	28,6	32,6	29,3	29,3
H770					42,3	36,5			34,4		38,2	31,8	31,4	34,0	31,2	37,5	28,0	29,0	27,8	28,6
H891		34,8				34,3			33,7		38,7	30,7	28,4	26,6	29,0	31,4	26,5	25,1	28,6	28,4
NTC						36,2			39,9		36,9	31,5	31,1	32,8	33,8	35,9	28,6	32,2	29,4	29,6

NTC, no-target control.

Supplemental Table 3. Calibration curve parameters for all the samples.

Field	Samples applied to	Treat.	Marker-filter	Error	Eff.	LOD [cop/gr]	LOQ [cop/gr]
Avinyó Nou	All	WP	FAM	0,18	2,14	2,57E+03	2,57E+03
			Cy5	0,14	2,16	2,63E+02	2,57E+03
			R610	0,01	2,09	2,63E+02	2,63E+02
		OD	FAM	0,15	2,12	1,04E+04	1,04E+04
			Cy5	0,00	2,21	1,04E+04	1,05E+05
			R610	0,01	1,83	1,04E+04	1,04E+04
		MS	FAM	0,11	2,40	6,05E+06	6,05E+06
			Cy5	0,03	2,39	6,05E+06	6,05E+06
			R610	0,01	2,40	6,05E+06	6,05E+06
Mont-roig del Camp*	11/27/19-03/04/20	WP	FAM	0,02	1,88	3,75E+00	3,67E+03
			Cy5	0,15	1,88	3,75E+00	3,75E+00
			R610	0,10	1,88	3,75E+00	3,61E+02
		OD	FAM	0,06	1,90	2,97E+00	2,89E+03
			Cy5	0,05	2,05	2,94E+01	2,89E+03
			R610	0,08	1,87	2,81E+02	2,89E+03
		MS	FAM	0,00	1,57	1,63E+03	1,63E+03
			Cy5	0,60	1,82	1,63E+03	1,63E+03
			R610	0,18	1,63	1,60E+02	1,63E+03
	03/30/20-07/29/20	WP	FAM	0,17	1,93	3,61E+02	3,61E+02
			Cy5	0,17	1,94	3,59E+01	3,59E+01
			R610	0,18	1,93	3,61E+02	3,61E+02
		OD	FAM	0,08	1,80	1,47E+04	1,47E+04
			Cy5	0,06	1,86	1,41E+02	1,41E+02
			R610	0,15	1,99	1,46E+03	1,46E+03
		MS	FAM	0,00	1,73	8,52E+07	8,52E+07
			Cy5	0,00	1,60	8,52E+07	8,52E+07
			R610	0,00	1,64	8,52E+07	8,52E+07
Caldes de Montbui I	All	OD	FAM	0,21	1,71	1,81E+03	1,81E+03
			Cy5	0,16	1,77	1,81E+03	1,81E+03
			R610	0,20	1,74	1,81E+03	1,81E+03
		OD	FAM	0,18	1,73	1,98E+02	1,98E+02
			Cy5	0,15	1,72	1,98E+02	1,98E+02
			R610	0,10	1,74	1,98E+02	1,98E+02
Caldes de Montbui II	All	OD	FAM	0,19	1,72	1,84E+02	1,81E+03
			Cy5	0,09	1,71	1,81E+03	1,80E+04
			R610	0,17	1,81	1,81E+03	1,81E+03
		OD	FAM	0,11	1,95	1,39E+03	1,39E+03
			Cy5	0,14	1,86	1,35E+02	1,39E+03
			R610	0,19	1,87	1,39E+03	1,39E+03

*Two calibration curves were needed for the samples from Mont-roig del Camp.

Supplemental Table 4. Spearman's correlation coefficients (ρ) of *Metarhizium brunneum* EAMb 09/01-Su concentration with different weather parameters. Boldface indicates statistically significant correlation ($P < 0.05$).

Trial	Prot.	Marker	PPT	TM	TX	TN	HRM	RS24
Avinyó Nou	WP	FAM	0,205	0,154	0,247	0,027	-0,039	0,416
		CY5	-0,105	-0,107	-0,107	-0,150	-0,342	0,266
		R610	-0,166	-0,186	-0,186	-0,227	-0,313	0,270
	OD	FAM	0,294	-0,168	-0,168	-0,191	-0,341	0,475
		CY5	0,279	-0,114	-0,114	-0,147	-0,283	0,491
		R610	0,202	-0,255	-0,255	-0,278	-0,382	0,318
	MS	FAM	-0,280	-0,218	-0,218	-0,217	-0,028	-0,056
		CY5	-0,211	0,018	0,018	0,033	0,242	-0,127
		R610	-0,343	-0,047	-0,047	-0,073	0,398	-0,449
Mont-roig del Camp	WP	FAM	0,184	-0,099	-0,095	-0,096	-0,210	-0,049
		CY5	N/A	N/A	N/A	N/A	N/A	N/A
		R610	0,247	0,043	0,024	0,073	-0,201	0,100
	OD	FAM	0,129	0,203	0,216	0,262	-0,198	0,234
		CY5	N/A	-0,025	0,042	-0,017	0,066	0,160
		R610	N/A	-0,018	0,021	-0,039	-0,065	0,282
	MS	FAM	N/A	0,017	0,014	0,019	-0,068	0,280
		CY5	N/A	0,756	0,756	0,756	-0,756	N/A
		R610	N/A	-0,093	-0,079	0,130	-0,149	0,354
Caldes de Montbui I	OD	FAM	-0,291	-0,350	-0,351	-0,197	0,249	-0,253
		CY5	-0,112	-0,355	-0,357	-0,241	0,333	-0,306
		R610	N/A	-0,418	-0,412	0,236	0,201	-0,306
Caldes de Montbui II	OD	FAM	-0,154	-0,820	-0,213	-0,820	0,213	-0,213
		CY5	-0,038	0,129	0,003	0,089	0,206	-0,039
		R610	-0,127	0,221	0,106	0,169	0,132	-0,011

Rain, rainfall (mm); TM, TX and TN, daily average, maximum and minimum temperatures, respectively (ºC); HRM, daily average relative humidity (%); RS24, daily accumulated solar radiation ($MJ \cdot m^{-2}$).
